# Lamin A, Chromatin and FPLD2: Not Just a Peripheral *Ménage-à-Trois*

**DOI:** 10.3389/fcell.2018.00073

**Published:** 2018-07-09

**Authors:** Nolwenn Briand, Inswasti Cahyani, Julia Madsen-Østerbye, Jonas Paulsen, Torunn Rønningen, Anita L. Sørensen, Philippe Collas

**Affiliations:** ^1^Department of Molecular Medicine, Faculty of Medicine, Institute of Basic Medical Sciences, University of Oslo, Oslo, Norway; ^2^Department of Immunology and Transfusion Medicine, Norwegian Center for Stem Cell Research, Oslo University Hospital, Oslo, Norway

**Keywords:** adipose stem cell, chromatin, FPLD2, lamin A, lipodystrophy, miR-335, T box

## Abstract

At the nuclear periphery, the genome is anchored to A- and B-type nuclear lamins in the form of heterochromatic lamina-associated domains. A-type lamins also associate with chromatin in the nuclear interior, away from the peripheral nuclear lamina. This nucleoplasmic lamin A environment tends to be euchromatic, suggesting distinct roles of lamin A in the regulation of gene expression in peripheral and more central regions of the nucleus. The hot-spot lamin A R482W mutation causing familial partial lipodystrophy of Dunnigan-type (FPLD2), affects lamin A association with chromatin at the nuclear periphery and in the nuclear interior, and is associated with 3-dimensional (3D) rearrangements of chromatin. Here, we highlight features of nuclear lamin association with the genome at the nuclear periphery and in the nuclear interior. We address recent data showing a rewiring of such interactions in cells from FPLD2 patients, and in adipose progenitor and induced pluripotent stem cell models of FPLD2. We discuss associated epigenetic and genome conformation changes elicited by the lamin A R482W mutation at the gene level. The findings argue that the mutation adversely impacts both global and local genome architecture throughout the nucleus space. The results, together with emerging new computational modeling tools, mark the start of a new era in our understanding of the 3D genomics of laminopathies.

## Introduction

### A short tale of LADs

**Figure 1:**
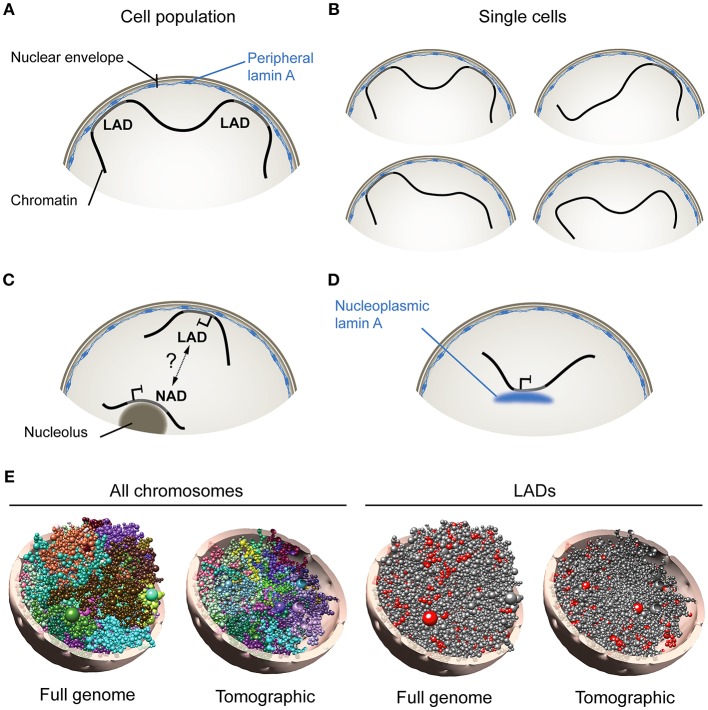
Association of A-type lamins with chromatin at the nuclear periphery and in the nuclear interior. **(A)** Lamina-associated domains (LADs), at the nuclear envelope, schematized from analyses of cell populations using genome-wide approaches such as DamID- or ChIP-sequencing. **(B)** Analysis of single cells however (e.g., by FISH) indicates that not all LADs mapped in cell populations are found at the nuclear periphery in all cells. This suggests that LADs constitute domains that dynamically anchor to, or detach from, the nuclear lamina. **(C)** Nucleolus-associated domain (NAD). How NADs arise remains uncertain, but may involve interchangeability of heterochromatic domains between the nuclear envelope and nucleolar borders. **(D)** Nucleoplasmic lamin A interacts with chromatin in the nuclear interior. (**E**, Left) Computational 3D model of the genome in a diploid human fibroblast nucleus taking into account genome-wide chromosomal interactions and interactions between chromatin and the nuclear periphery. Models show all chromosomes in whole-genome and tomographic representations. Each chromosome is differently colored and modeled as a chain of beads, each bead representing a topological domain determined from chromosome conformation capture (Hi-C) data. Right: Positioning of lamin A LADs (red beads) is shown. Radial placement of beads is determined from, here, lamin A ChIP-sequencing data in fibroblasts. 3D genome models were constructed using Chrom3D (Paulsen et al., 2017).

The periphery of the mammalian nucleus is delineated by the nuclear envelope and by subjacent domains of compact and repressed heterochromatin separated by more open and active regions in the vicinity of nuclear pores. The nuclear envelope consists of an outer and inner membrane, nuclear pores and the nuclear lamina, a polymer of A-type lamins (lamin A/C, abbreviated here as lamin A) and B-type lamins (lamins B1 and B2) (Burke and Stewart, 2013). While some integral proteins of the inner nuclear membrane are able to interact with chromatin, the best characterized interactions of the nuclear envelope with the genome are mediated by lamins and so-called lamina-associated domains (LADs) (van Steensel and Belmont, 2017; Figure 1A). Evidence for LADs has been provided by Dam methylase identification (DamID) of nuclear lamin-associated DNA regions (Guelen et al., 2008) and by chromatin immunoprecipitation (ChIP) of lamins (Lund et al., 2014), followed by sequencing of the associated DNA. With these techniques, individual LADs

have been shown to range from ~0.1 to 10 megabases and altogether make up 25–30% of the genome (Meuleman et al., 2013). LADs are typically AT-rich, gene-poor and heterochromatic, and accordingly, most genes found in LADs are repressed or expressed at low levels (Guelen et al., 2008; Reddy et al., 2008). The position of LADs along the linear genome is overall conserved between cell types (Meuleman et al., 2013). However, more variable LADs also exist, which show less consistency between cell types or during differentiation, and are often smaller than constitutive LADs (Meuleman et al., 2013; Rønningen et al., 2015; Gesson et al., 2016).

The patterns of nuclear lamin association with the genome can deviate from “classical LADs” found at the nuclear periphery. Moreover, disease-causing mutations in lamin A can alter lamin-chromatin interactions, not only at the nuclear periphery, but also in the nuclear center. Recent findings discussed here imply an impact of lamin A mutations on chromatin architecture globally and at the gene level.

### Lamin-chromatin interactions at the nuclear periphery and in the nuclear interior

At the nuclear periphery, increasing evidence suggests that chromatin represents as a dynamic compartment. Accordingly, fluorescence *in situ* hybridization (FISH) data show that not all lamin B1 LADs mapped by DamID from cell populations are found at the nuclear periphery in individual cells (Kind et al., 2013, 2015). This also holds true for LADs mapped by ChIP (Paulsen et al., 2017). Therefore, sequences making up LADs can be found both at the nuclear periphery and in the nuclear center at the single-cell level. Thus, LADs represent genomic regions that interact with the nuclear lamina when mapped from cell populations using high-throughput genomic techniques (Figure 1A), yet they may dynamically anchor at, and detach from, the nuclear lamina in single cells, providing a variegated assortment of lamin-genome associations (Figure 1B).

In the nuclear interior, chromatin can also associate with nucleoli in the form of nucleolus-associated domains (NADs) (Németh et al., 2010; van Koningsbruggen et al., 2010). Intriguingly, LADs and NADs share similarities in size, AT content, gene density and heterochromatic features. Thus NADs and LADs may perhaps represent interchangeable domains resulting from a displacement of heterochromatin away from, or toward, the nuclear periphery (van Koningsbruggen et al., 2010; Figure 1C). NADs could also result from nuclear envelope invaginations (Fricker et al., 1997) apposing peripheral heterochromatin to nucleoli. A transient peripheral localization of nucleoli has also been observed after depletion of lamin B1 (Martin et al., 2009), but even though lamins can biochemically co-fractionate with nucleoli (Martin et al., 2009), there is still no compelling evidence of lamin association with these structures. The relationship between NADs and LADs, and mechanisms of how these arise, remain therefore to be investigated.

Whereas B-type lamins are restricted to the nuclear envelope, A-type lamins also exist as a nucleoplasmic pool (Kolb et al., 2011) able to associate with chromatin (Gesson et al., 2016) (Figure 1D). Remarkably, intranuclear lamin A LADs can display features of euchromatin (Lund et al., 2015; Gesson et al., 2016); binding of lamin A to these euchromatic regions is dependent on the lamin A-interacting partner lamina-associated polypeptide LAP2α, which is strictly nucleoplasmic and not enriched at the nuclear periphery (Gesson et al., 2016). The nucleoplasmic lamin A pool is notably required for the stabilization of Polycomb bodies in the nucleus, and for the maintenance of the repressive function of Polycomb at proper target genes (Cesarini et al., 2015; Marullo et al., 2016). These observations imply that, notwithstanding the fact that LADs can be released from the nuclear lamina (and as such lose their “LAD” annotation), LADs, as *lamin*-associated domains, can be found both at the nuclear periphery and in the nuclear interior.

These implications raise the issue of whether there is unambiguous evidence of constitutive LADs being associated with nucleoplasmic lamin A (in the nucleus center) vs. peripheral lamin A. To our knowledge, the short answer is at present “no.” (i) The soluble fraction of nucleoplasmic lamin A is probably unlikely to specifically associate with chromatin (this is still unknown). (ii) The chromatin-associated fraction of nucleoplasmic lamin A is not unequivocally distinguishable from the peripheral (lamina-associated) fraction of lamin A by high-throughput genomics techniques (ChIP or DamID). Implications have been derived from ChIP-sequencing data, suggesting that LADs with a higher gene density and with more euchromatic features than *bona fide* (peripheral) LADs would be found in the nuclear interior (Lund et al., 2015), but this is no proof. (iii) Association of chromatin-bound LAP2α with lamin A, in the nuclear interior, would provide an opportunity to distinguish between peripheral and internal lamin A LADs, through a differential analysis of LADs identified in cells containing LAP2α or depleted of LAP2α (Gesson et al., 2016). Since nucleoplasmic lamin A relocalizes to the nuclear periphery in LAP2α knock-out cells (Gesson et al., 2016), one could speculate that at least a fraction of LADs specific to wild-type cells might predominantly be intranuclear. However, LADs would also likely be reorganized at the nuclear periphery in LAP2α knock-outs, making compelling arguments difficult. Moreover, these experiments would not address the question of *constitutive* lamin A LADs exist in the nuclear center. (iv) Single-cell imaging approaches such as immuno-FISH might help evaluate the proximity of a given LAD to nucleoplasmic lamin A across cells in a population, albeit not with the resolution required to clearly define this region as a constitutive LAD. The current data therefore suggest the possibility of a dynamic exchange of LADs between the nuclear periphery and the nuclear interior. Live-cell imaging experiments tracking labeled LADs in individual cells throughout the cell cycle or after various perturbations might provide new insights on the intranuclear dynamics of LADs.

The cell-to-cell variability in the radial distribution of LADs observed by FISH can interestingly be predicted by computational 3-dimensional (3D) whole genome modeling techniques that take into account genome-wide chromosomal interactions and LAD information (Li et al., 2017; Paulsen et al., 2017, 2018; Figure 1E; shown here for one genome model). Analysis of hundreds of 3D genome models can provide information on e.g., the composition of LADs as a function of their distance to the nuclear periphery (Paulsen et al., 2017). As addressed below, 3D genome models have also proven useful to predict alterations in spatial genome conformation in cells expressing lamin A mutations linked to laminopathies (Paulsen et al., 2017).

### FPLD2-causing lamin A R482W mutation: a poster child for rearrangement of lamin-chromatin interactions

Nearly 500 mutations across the *LMNA* gene, which encodes lamin A/C, have been linked to diseases commonly referred to as laminopathies (Worman and Schirmer, 2015). These include muscle dystrophies, cardiomyopathies, peripheral neuropathies, premature aging, and of particular interest for the work outlined here, partial lipodystrophies (Cao and Hegele, 2000; Hegele et al., 2000; Shackleton et al., 2000; Vigouroux et al., 2011; Dobrzynska et al., 2016). The heterozygous lamin A p.Arg482Trp (R482W) substitution (Cao and Hegele, 2000; Shackleton et al., 2000) has become a poster child of lipodystrophic laminopathies because it is the most frequent lamin A mutation underlying these particular diseases. The mutation causes familial partial lipodystrophy of Dunnigan type (FPLD2), characterized by adipose tissue atrophy in the lower limbs, visceral and cervical fat accumulation, muscle hypertrophy and severe metabolic disorders (Decaudain et al., 2007; Guenantin et al., 2014). FPLD2 patients also show early-onset atherosclerosis leading to cardiovascular pathologies (Garg, 2000; Hegele, 2001; Bidault et al., 2013). In cellular models of FPLD2, the R482W mutation leads to deficiencies in adipogenesis (Oldenburg et al., 2014, 2017) and in mesodermal and endothelial differentiation (Briand et al., 2018). The mutation also involves defects in nuclear morphology (Vigouroux et al., 2001), adipogenic transcription factor compartmentalization (Vadrot et al., 2014) and signal transduction (Le Dour et al., 2017).

The R482W mutation lies in the immunoglobulin fold of lamin A, but does not severely disrupt the structure of the fold because the R482 residue lies on its surface. However, the mutation impairs interaction of the immunoglobulin fold with DNA and nucleosomes *in vitro* (Stierle et al., 2003). Because lamin A is key factor in the radial (that is, periphery vs. center) distribution of euchromatin and heterochromatin (Solovei et al., 2013) and in constraining chromatin movement within the nucleus (Bronshtein et al., 2015), the R482W mutation may perturb associations of lamin A with chromatin, and thereby spatial genome conformation.

### Lamin A R482W rearranges peripheral and intranuclear LADs

Recent studies point to the FPLD2 lamin A R482W mutation as a trigger of abnormalities in chromatin architecture on a large scale and at the gene level. These anomalies do not strictly localize at the nuclear periphery: they also affect important developmental genes in the nuclear interior (Oldenburg et al., 2017; Paulsen et al., 2017; Briand et al., 2018). We have recently shown that lamin A LADs mapped in fibroblasts from FPLD2 patients with the R482W mutation and from controls with wild-type lamin A are overall conserved; yet some LADs differ between patients and controls (Paulsen et al., 2017). Moreover, computational 3D genome modeling enables predictions of the impact of lamin mutations on genome organization in the 3D nucleus.

Unsuspected features of lamin A-chromatin associations have emerged from analyses of 3D genome models of FPLD2 and control fibroblasts (Paulsen et al., 2017). First, LADs specific to FPLD2 patient fibroblasts occupy more central positions than all LADs in these cells. In contrast, LADs unique to controls are enriched at the periphery in these cells; interestingly, these regions are repositioned toward the nuclear interior in FPLD2 patient cells, being free of lamin A. Further, gained or lost LADs in patient cells harbor genes important for adipocyte function and metabolism (Paulsen et al., 2017), raising the hypothesis that deregulation of positioning and/or expression of these genes in cells with the lamin A R482W mutation may be relevant for the phenotypes characterizing the disease.

These findings argue that analyzing genomic features with a 3D perspective unveils unsuspected alterations in large-scale genome architecture, not only the nuclear periphery but also in the nuclear interior, by the lamin A R482W mutation. Whether this holds true for other mutations in the immunoglobulin fold of lamin A remains a possibility that needs to be explored. A recent investigation of LADs associated with overexpressed wild-type or mutant lamin A, including lamin A R482W, intriguingly argues for dramatic LAD differences (Perovanovic et al., 2016). However, these seem incompatible with the overall LAD similarity reported in FPLD2 patient cells with the R482W mutation (Paulsen et al., 2017); they and could be due to the overexpressed nature of lamin A in the former study, and to discrepancies in the algorithms used to map LADs in both studies (Lund et al., 2014).

### Outside LADs (part 1): deregulation of a developmentally-regulated micro-RNA gene in an adipose stem cell model of FPLD2

In addition to altering the distribution of LADs in the 3D nucleus, the lamin A R482W mutation more locally affects the conformation of loci important for differentiation. We have found that expressing the mutation in human adipose stem cells (ASCs) impairs activation of adipogenic genes and differentiation into adipocytes *in vitro* (Oldenburg et al., 2014). This co-occurs with the maintenance of an anti-adipogenic micro-RNA, miR-335, in an overexpressed state (Oldenburg et al., 2017). Bringing miR-335 back to baseline levels with a specific inhibitor rescues adipogenic gene expression. Thus, lamin A R482W imposes a dominant anti-adipogenic phenotype in an ASC model of FPLD2 via deregulation of an anti-adipogenic micro-RNA. Whether this in turns affects expression of mRNAs targeted by miR-335 remains to be examined.

**Figure 2:**
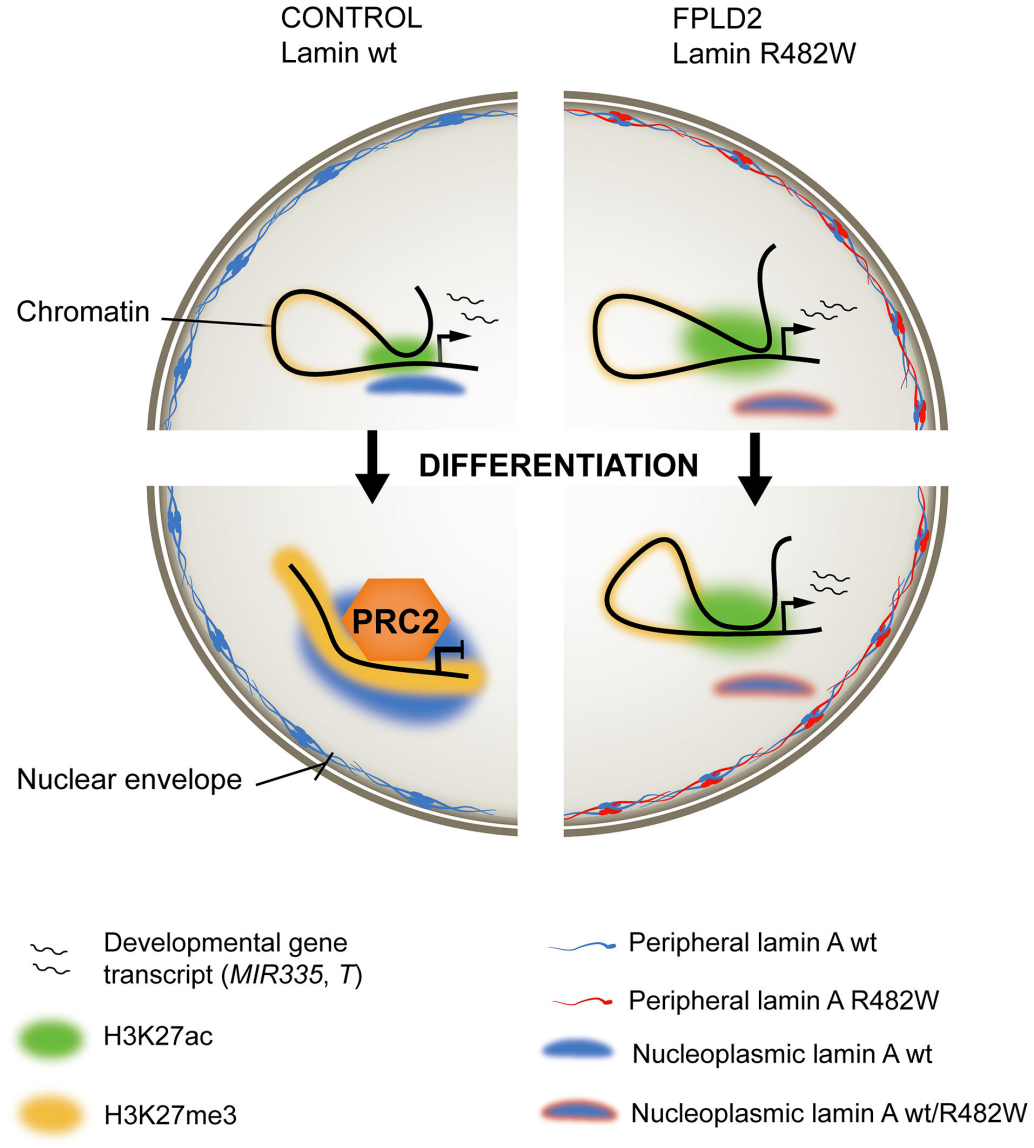
*Ménage-à-trois* between lamin A, chromatin and FPLD2. **(Left)** In wild-type (wt) stem or progenitor cells, an active developmentally-regulated gene (top) becomes repressed and maintained inactive by the PRC2 repressor complex after induction of differentiation (bottom). Active regulatory regions, H3K27 acetylated (H3K27ac) in the undifferentiated state, become H3K27 methylated (H3K27me3). Wild-type lamin A associates with the locus, possibly stabilizing a repressed chromatin conformation. **(Right)** In stem or progenitor cells expressing the heterozygous lamin A R482W mutation, the mutation imposes a dominant-negative effect on lamin A binding to the locus, PRC2 targeting and H3K27 methylation. This correlates with maintenance of gene expression (exemplified by the *MIR335* gene in an adipose stem cell model of FPLD2), or delayed transcriptional inactivation (exemplified by the *T/BRACHYURY* gene in an iPS cell model of FPLD2).

The mechanisms of deregulation of *MIR335* expression by the lamin A mutation appear to entail structural, conformational and epigenetic components (Oldenburg et al., 2017). First, the R482W mutation exerts an adverse effect on lamin A binding to the *MIR335* locus, which normally occurs during differentiation of wild-type cells (Figure 2). Considering the repressive role of lamin A on gene expression (Lund et al., 2013), this dominant-negative phenotype is consistent with overexpression of *MIR335* in mutant cells. Second, the mutation abolishes repositioning of the *MIR335* locus, identified by FISH in the nuclear interior, toward the more repressive nuclear periphery, which normally occurs in differentiating wild-type ASCs. This suggests an involvement of nucleoplasmic lamin A in the regulation of 3D positioning of loci important for ASC differentiation. Third, defective lamin A binding to *MIR335* in lamin A mutant ASCs coincides with an absence of repressive trimethylated histone H3 lysine 27 (H3K27me3) marking by Polycomb across the *MIR335* promoter and enhancers; these regulatory elements are instead H3K27 acetylated (Figure 2). This observation is reminiscent of the mis-localization of the Polycomb repressor complex PCR2 in myoblasts depleted of lamin A, ectopic H3K27me3 and deregulation of myogenic gene expression in these cells (Cesarini et al., 2015; Marullo et al., 2016). Lastly, dual-color FISH data suggests that the mutation favors *MIR335* enhancer-promoter proximity, which together with the H3K27 acetylated state of the enhancers again concurs with *MIR335* overexpression. The R482W mutation therefore elicits a conformational remodeling of the *MIR335* locus during differentiation induction, not at the nuclear periphery, but in the nuclear interior, preventing Polycomb-mediated repression of the gene after adipogenic induction (Figure 2).

### Outside LADs (part 2): temporal epigenetic deregulation of the mesodermal inducer *T/Brachyury* in an iPS cell model of FPLD2

The lipodystrophic syndrome of FPLD2 patients is complicated by severe and early-onset atherosclerosis leading to premature coronary heart disease, peripheral arteritis, and stroke (Garg, 2000; Hegele, 2001; Bidault et al., 2013). Expression of lamin A R482W in mature endothelial cells elicits cell dysfunction (Bidault et al., 2013), but using mature cells does not account for any developmental aspect of the disease. Interestingly, 3D genome models of fibroblasts from several FPLD2 patients with the lamin A R482W mutation illustrate a repositioning of the *T/BRACHYURY* gene (Briand et al., 2018), a key regulator of mesodermal differentiation (Garnett et al., 2009; Turner et al., 2014; Beisaw et al., 2018), toward the nuclear center relative to wild-type cells. This displacement of *T* in FPLD2 fibroblasts correlates with a lack of lamin A binding to the locus in these cells, whereas strikingly, *T* lies at a lamin A LAD border (and is bound by lamin A) in wild-type cells (Briand et al., 2018). Since the nuclear center is more permissive to gene activity than the periphery, these models raise the hypothesis of enhanced *T* activation potential in a developmental model of FPLD2.

Addressing this issue, Briand, Guénantin and colleagues have in a collaborative effort generated induced pluripotent stem (iPS) cells from a patient with FPLD2 linked to the lamin A R482W mutation, and genetically corrected the *LMNA* c.1444C > T substitution to generate isogenic *LMNA* wild-type iPS cells (Briand et al., 2018). Endothelial differentiation of iPS cells through consecutive steps of mesodermal induction and endothelial specification shows transcriptional and phenotypic defects associated with the mutation. More specifically, transcriptome data link differentiation anomalies to precocious and overexpression of *T* and of T-box target genes, many of which are implicated in endothelial differentiation. Again, *T* overexpression concurs with defective PRC2 anchoring to the *T* locus and failure to mediate H3K27 methylation to repress *T* in a timely fashion (Figure 2). Similarly to *MIR335* deregulation in ASCs, this lack of timely *T* inactivation correlates with impaired lamin A association with the *T* locus in mutant iPS cells (Briand et al., 2018).

Observations reported here link the FPLD2-causing lamin A R482W mutation to a deregulation of adipogenic and mesodermal and endothelial gene expression in somatic and pluripotent stem cell models of the disease. Chromatin remodeling events at the affected loci take place in the nuclear interior, underscoring the broad impact of lamin A on chromatin conformation. The data suggest that the lamin A R482W mutation impinges on chromatin architecture throughout the 3D nucleus, altering the fate of mesodermal lineages, and plausibly underlining the multiple tissue phenotypes of FPLD2.

## Concluding remarks

The recent findings discussed here illustrate a *ménage-à-trois* between lamin A, chromatin and FPLD2. In this three-way relationship, the FPLD2 disease status imposes a dominant-negative deregulation of chromatin architecture, which involves lamin A through the R482W mutation. The results reveal a remodeling of lamin-genome associations in the 3D nucleus, both at the periphery and in the center. In both adipose and iPS cell models of the disease, these perturbations implicate the Polycomb repressor complex and a failure to repress genes in a scheduled manner. This in turn leads to, in the examples provided, a deregulation of adipose and mesodermal/endothelial cell fates.

Future studies will need to address how wide-spread this lack-of-repression phenotype imposed by the FPLD2 lamin A mutation is across the genome in tissue-specific stem cells. The immunoglobulin fold of lamin A interacts with chromatin and there are at least two accounts of mutations in this region which affect lamin-chromatin interactions genome-wide (Perovanovic et al., 2016; Paulsen et al., 2017), yet this does not exclude the possibility that mutations elsewhere in the lamin A protein also alter lamin-genome interactions. The impact of other lamin A mutations causing laminopathies, such as lipodystrophies, muscle dystrophies, peripheral neuropathies or progeria, on LADs and genome organization remains to be examined. An important question is to what extent lamin A mutations affecting specific tissues deregulate chromatin conformation, epigenetic states and gene expression at specific loci important for differentiation and homeostasis of these tissues and not others. Genomic deregulations imposed by lamin A mutations may be underpinned by protein factors mediating these effects in a cell or tissue type- and locus-specific manner. Proteins implicated in the anchoring of chromatin to nuclear lamins and/or targeting specific loci to the nuclear lamina (at the nuclear periphery) are the subject of investigations (Zullo et al., 2012; Shachar et al., 2015), but remain challenging to identify. Combinations of interactome proteomics aiming to characterize cell type-specific factors associated with wild-type or mutant lamin A (e.g., Kubben et al., 2010; Oldenburg et al., 2014; Bar et al., 2018), and genomic mapping of these complexes, in well-chosen cellular models of laminopathies will constitute important experimental next steps in our molecular understanding of these diseases. Lastly, spatial investigations of genome conformation through computational 3D modeling of chromatin are expected to generate new testable hypotheses in upcoming studies of the genomics of laminopathies and other pathologies where disease-causing factors are susceptible to differentially affect the genome in a spatial manner (García-Nieto et al., 2017).

## Author contributions

NB: conception, writing, figures; IC, TR, and AS: conception; JM-Ø: conception, writing; JP: conception, figures; PC: conception, writing, supervision.

### Conflict of interest statement

The authors declare that the research was conducted in the absence of any commercial or financial relationships that could be construed as a potential conflict of interest.

## Abbreviations

ASC: adipose stem cell
ChIP: chromatin immunoprecipitation
DamID: Dam methylase identification
FISH: fluorescence *in situ* hybridization
FPLD2: familial partial lipodystrophy of Dunnigan-type
iPSC: induced pluripotent stem cell
LAD: lamina-associated domain.
